# Development and validation of a healthy lifestyle and behavioral score for predicting metabolic syndrome: evidence from a 24-year longitudinal health check-up cohort in Taiwan

**DOI:** 10.1186/s12966-026-01940-x

**Published:** 2026-06-18

**Authors:** Jansen M. Cambia, Ta-Chien Chan

**Affiliations:** 1 Research Center for Humanities and Social Sciences, Academia Sinica, 128 Academia Road, Section 2, Nangang, Taipei, 115 Taiwan; 2 Institute of Public Health, School of Medicine, National Yang Ming Chiao Tung University, Taipei, Taiwan; 3 Department of Public Health, College of Public Health, China Medical University, Taichung Campus, Taichung, Taiwan; 4 School of Medicine, College of Medicine, National Sun Yat-sen University, Kaohsiung, Taiwan

**Keywords:** Behavioral risk factors, Healthy lifestyle, Longitudinal cohort, Metabolic syndrome

## Abstract

**Background:**

Metabolic syndrome (MetS) is a multifactorial condition characterized by obesity, hypertension, hyperglycemia, and dyslipidemia, often resulting from unhealthy lifestyle and behavioral patterns. This study aimed to develop and validate a Healthy Lifestyle and Behavioral Scale (HLBS) that reflects the combined influence of key pillars of lifestyle medicine and to examine its longitudinal association with MetS risk in a large Taiwanese cohort.

**Methods:**

Data were drawn from 201,123 participants of the Mei Jau Health Management Institution between 2000 and 2023. Item selection followed a rigorous construct validation process and selection criteria, including exploratory and confirmatory factor analyses, item response theory modeling, and reliability testing. We then combined the HLBS components such as diet, physical activity, sleep, and substance use, and performed a log binomial generalized estimating equation model to assess its risk association with MetS development.

**Results:**

The HLBS demonstrated acceptable model fit and strong internal consistency. Moderate (RR = 1.12; 95% CI = 1.09–1.16) and low (RR = 1.46; 95% CI = 1.41–1.50) adherence to healthy lifestyle behaviors were associated with an increased risk of developing MetS compared with high adherence, and each HLBS component independently contributed to elevate MetS risk. Stratified analyses showed consistent linear associations across all age and sex groups, with stronger effects observed among adults and the elderly.

**Conclusions:**

The HLBS provides a reliable, culturally adapted tool for assessing lifestyle behaviors and identifying populations at risk for metabolic disorders. These findings emphasize the importance of promoting comprehensive lifestyle interventions to reduce MetS risk in the general population.

**Supplementary Information:**

The online version contains supplementary material available at 10.1186/s12966-026-01940-x.

## Background

Metabolic syndrome (MetS) is a cluster of physiological risk factors characterized by abdominal obesity, hypertension, hyperglycemia, hypertriglyceridemia, and reduced levels of high-density lipoprotein cholesterol (HDL-C) [1]. Prevalence estimates vary by diagnostic criteria, with a meta-analysis reporting a global range of 12.5%–31.4% [2]. MetS is a major risk factor for chronic diseases such as cardiovascular disease, cancer, and cognitive decline [3].

The pathogenesis of MetS is multifactorial, involving nutritional, environmental, and genetic factors, with lipid metabolism dysregulation, insulin resistance, and chronic inflammation playing central roles [4]. Also, exposure to unhealthy lifestyle and behavioral factors, including sleep disturbance, suboptimal nutrition, physical inactivity, and substance use, along with population aging, are major contributors [4–7]. Lifestyle modification has been proposed as an effective strategy for preventing chronic diseases, including MetS [8]. While previous studies have identified individual risk factors [5–7], evidence suggests that multiple lifestyle risks should be considered together, as their combined effect is more detrimental than any single factor [9, 10]. Identifying high-risk groups can inform targeted health promotion strategies [11]. Most studies to date, including those examining single or combined lifestyle risks [5–7, 11, 12], have been cross-sectional, highlighting the need for longitudinal studies to better assess causal relationships in the general population [11].

The healthy lifestyle and behavioral scale (HLBS) is an evidence-based tool encompassing key lifestyle pillars, including sleep, nutrition, physical activity, and substance use [13, 14]. Grounded in health behavior change theories, including the transtheoretical model, self-determination theory, and the multiple behavior approach, which emphasizes simultaneous modification of multiple health behaviors to maximize overall health benefits [15, 16]. Through this framework, the HLBS promotes actionable behavior changes that can influence both physical and mental health [13]. The scale has been widely used to examine associations between lifestyle behaviors and various health conditions such as diabetes, cardiometabolic disorders, neuroendocrine dysfunction, and insulin resistance [11, 12, 17]. These conditions are key components of MetS and highlight the importance of comprehensive lifestyle assessment in this context, reinforcing the relevance of multi-behavior frameworks [18]. Previous studies have also employed questionnaires targeting individual HLBS components [11, 12]. However, Pangalangan (2024) noted that many validated instruments are limited in scope, addressing only certain HLBS pillars and failing to capture the full framework [13]. Moreover, many of these instruments were originally developed in Western contexts and subsequently adapted for use in HLBS measurement [15]. Thus far, few instruments have been specifically developed for lifestyle medicine, and even fewer have undergone rigorous scale development and validation, particularly some Asian populations. In this study, the HLBS is applied within a Taiwanese population, where its psychometric and behavioral relevance has been previously examined [19].

Given these identified gaps, this study aimed to develop a HLBS using data from a longitudinal cohort of Taiwanese health check-up institutions. Furthermore, we sought to examine the association between the developed HLBS and its individual components with the risk of developing MetS over time.

## Methods

### Study participants and selection process

The cohort was established by the Mei Jau (MJ) Health Management Institution and is maintained by the MJ Health Research Foundation, a private organization that provides comprehensive health screening for the Taiwanese population (http://www.mjhrf.org/index.php). Between 2000 and 2023, a total of 523,437 participants across all age groups were enrolled, with data collected on socioeconomic factors, behavioral characteristics, and physical examinations [20].

For the selection process, individuals with MetS were identified according to the Ministry of Health and Welfare, Taiwan (see Supplementary Fig. 1). The Taiwanese definition of MetS requires the presence of three or more of the following: (1) abdominal obesity: waist circumference ≥ 90 cm for men and ≥ 80 cm for women [21]; (2) hypertension: systolic BP (blood pressure) ≥ 130 mmHg or diastolic BP ≥ 85 mmHg (measured from both arms), as well as the use of antihypertensive drugs; (3) hyperglycemia: fasting plasma glucose (FPG ≥ 100 mg/dL) or current use of antihyperglycemic drugs [22]; (4) hypertriglyceridemia, triglyceride levels ≥ 150 mg/dL or use of triglyceride-lowering medication; and (5) low HDL-C < 40 mg/dL for men or < 50 mg/dL for women [23].

**Fig. 1:**
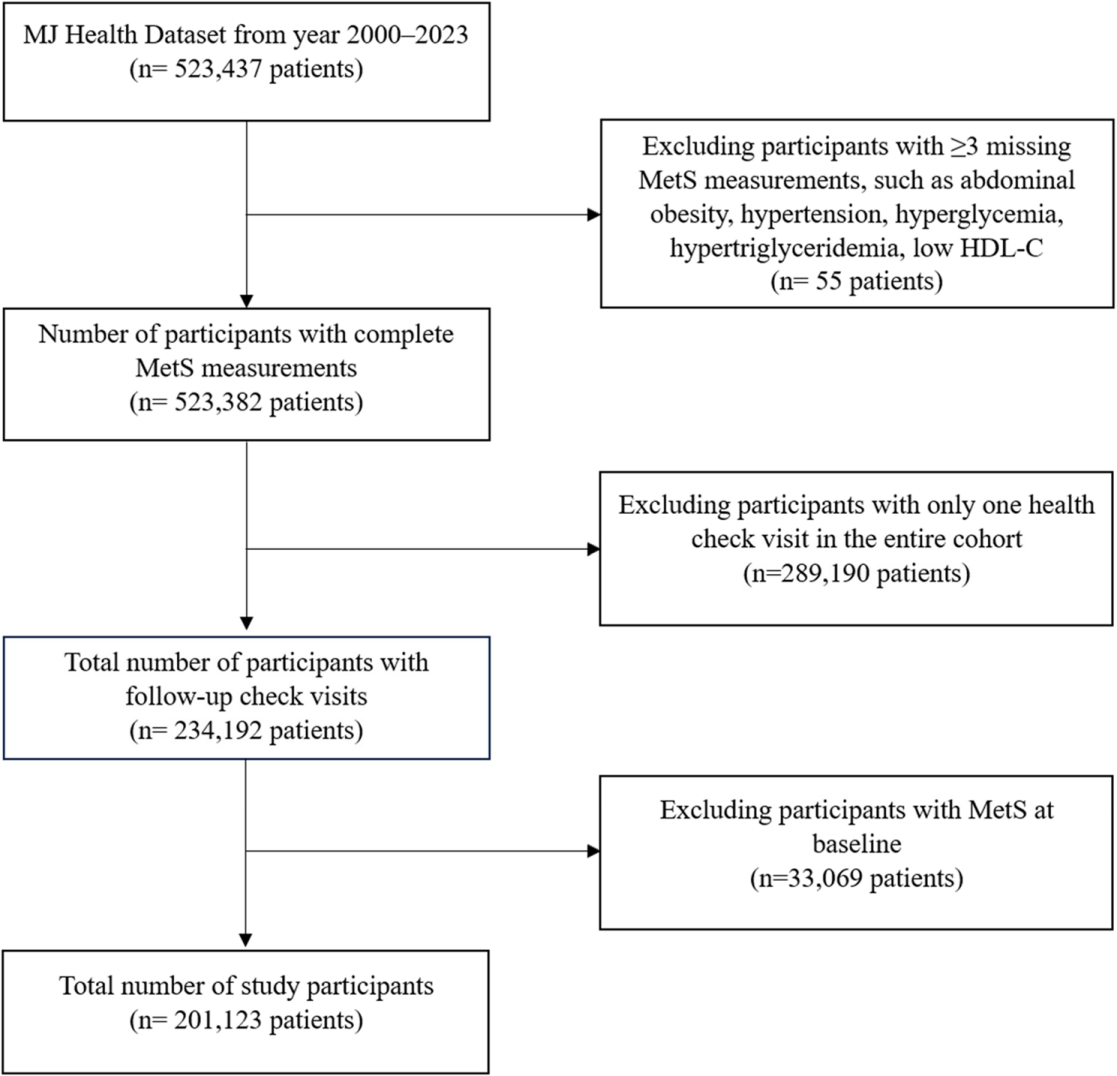
Flowchart of study inclusion criteria using the MJ Health Dataset

After identifying the total sample for MetS outcomes, the exclusion criteria for participants are outlined in Fig. 1. Participants entered the cohort at their first health check without MetS and were followed through subsequent visits until the first occurrence of MetS or their last available visit. Incident MetS was defined as the first occurrence of meeting MetS criteria among participants free of MetS at baseline. Participants with three or more missing MetS measurements in the baseline were excluded, as this made it impossible to classify their MetS status. Participants who could not be classified as having MetS due to missing outcome information at any point during the cohort period were excluded (*n* = 55 patients). In addition, those with only one health check visit in the entire cohort and those with MetS at baseline were excluded (*n* = 289,190 patients), as the aim was to identify new-onset MetS during follow-up. A detailed overview of exclusions and participant visits by year is provided in Supplementary Table 1.

Informed consent was obtained from all participants. The study was approved by the Institutional Review Board on Biomedical Science Research. All individually identifiable information was anonymized, and the study was conducted in accordance with the Declaration of Helsinki and the approved protocol.

### Selection criteria of the HLBS

We developed a HLBS to assess four components of the lifestyle medicine pillars, including sleep disturbance, physical inactivity, suboptimal nutrition, and substance use. This scale was adapted from the lifestyle medicine pillars proposed by Frates et al. (2021) [14], and has been utilized in multiple studies to formulate healthy lifestyle and behavior measures [11–13]. Details on the number of baseline items screened for construct validity are provided in Appendix a. The subsequent process of item selection and evaluation is described in detail below.

### Item construct and handling missing data

Certain questionnaire item responses corresponding to specific healthy lifestyle behaviors were modified over time to improve measurement precision. To ensure comparability across questionnaire waves, these items were harmonized by mapping the revised activity-specific responses onto the original categorical framework, aligning conceptually equivalent activity levels, and creating unified categories for analysis across all participants. For a detailed description of the mapping process, see Appendix b.

Then, the identified items were screened for content validity to ensure that they accurately measured the constructs of interest. We examined the representativeness of each item in capturing the overarching construct (pillar) based on evidence from previous literature [11–13] (see Appendix c). From the initial item set, we identified 8 items related to sleep, 6 to physical activity, 33 to nutrition, and 11 to substance use. The complete list of items is presented in Supplementary Table 2.

The distribution of samples with structural and intermittent missingness in our longitudinal open cohort is presented in Supplementary Table 3, where intermittent missingness occurred across health check visits. To minimize bias and retain statistical power in developing the HLBS, we employed strategies that integrate missing data handling within factor analytic frameworks (see Appendix d).

### Validity and reliability analysis

We conducted an exploratory factor analysis (EFA) to examine the structure of each scale component, using tetrachoric correlations for binary and polyserial correlations for ordinal items. Factor loadings were used to calculate the proportion of variance explained by each factor, reflecting its contribution to the overall construct [24]. See Appendix e for the EFA best-fit criteria.

Item performance was further evaluated using a one-factor item response theory (IRT) framework. Dichotomous items (e.g., sleep disturbance indicators such as difficulty falling asleep or use of sleep medication) were modeled using the two-parameter logistic model, while ordinal items (e.g., levels of physical activity, dietary patterns, and substance use behaviors) were analyzed using the graded response model. For domains with mixed item types, each item was modeled according to its response format within the same latent construct [25]. A detailed mapping of items and corresponding IRT models was provided in Supplementary Table 2.

We also conducted confirmatory factor analysis (CFA), specifying a single latent factor for each refined item set. The weighted least squares mean and variance adjusted estimator was applied to categorical and ordinal items, incorporating diagonal weighting with mean and variance adjustments [26, 27]. See Appendix e for CFA model-fit criteria.

Further, the internal consistency of each component was evaluated using Cronbach’s alpha, measuring how well items within a factor assess the same construct.

### Measuring the HLBS

We adopted the scoring criteria for the healthy lifestyle and behavior components from Pangalangan (2024), where each component was scored by summing the item values, with lower scores indicating healthier behaviors [13]. The score ranges for the components are sleep (0–5), physical activity (0–11), nutrition (0–40), and substance use (0–31). To construct the HLBS, adapted from previous studies [11, 17], we summed the component scores for each participant. The total HLBS was then categorized into tertiles (0–3), with higher scores indicating lower adherence and lower scores indicating higher adherence to healthy lifestyle behaviors.

Although IRT provides a framework for estimating latent trait scores, it was used in this study primarily to evaluate item performance and support item refinement rather than for score derivation [25]. Following psychometric validation using EFA, CFA, and IRT, all retained items demonstrated acceptable performance, supporting the assumption that each item contributed meaningfully to the construct. Therefore, an equal-weighted sum scoring approach was adopted. This approach enhanced interpretability and facilitated practical application in large-scale epidemiological and public health settings, where transparency and ease of implementation were essential [13]. Additionally, the use of summed scores was consistent with prior lifestyle indices, which commonly employed equal-weighted scoring to improve comparability and usability [13].

### Statistical analysis

We employed a Generalized Estimating Equations (GEE) approach to account for within-subject correlations arising from repeated measurements when examining the association between the HLBS and MetS across the longitudinal cohort. Although interval-censored survival models are commonly used and may also be appropriate for handling interval-censored event times, GEE was selected as it accommodates a binary per-visit outcome measured at irregular visit intervals [28]. Consequently, the estimated relative risks should be interpreted as visit-based risks rather than within a continuous-time risk framework used in standard time-to-event analyses.

A log-binomial GEE model was fitted using the “geepack” package in R (version 4.5.1) [29], specifying a binomial distribution with a log link to directly estimate risk ratios (RR) and 95% confidence intervals (CI). An exchangeable working correlation structure was assumed, indicating equal correlations between repeated observations from the same participant. The irregular timing of health check visits in this open cohort, RR from the GEE models correspond to the per-visit risk of incident MetS. To further assess whether the association between HLBS and metabolic risk differs across baseline metabolic status, we fitted a GEE model with a Gaussian distribution. To assess effect modification by baseline metabolic status, the model included an interaction term between HLBS and baseline metabolic risk group (1, 2, and ≥ 3 baseline components).

All models included the HLBS as the main predictor and were adjusted for demographic factors, including age at visit (< 20, 20–39, 40–59, ≥ 60 years), calendar years (2000–2023), sex (male, female), education (no formal education, elementary, junior high, high/vocational school, associate, bachelor’s, graduate or higher), and household annual income (quartile 1 = < 400,000 NTD; quartile 2 = 410,000–800,000 NTD; quartile 3 = 410,000–800,000 NTD; quartile 4 = 810,000–1,200,000 NTD; quartile 5 = 1,210,000–1,600,000 NTD; quartile 6 = 1,610,000–2,000,000 NTD; quartile 7 = > 2,010,000 NTD). For the analysis of each HLBS component, we adjusted for the demographic factors and included the other HLBS components (sleep quality, physical activity, nutritional intake, and substance use), excluding the component under analysis in the model. Analyses were further stratified by age and sex to explore potential effect modification across demographic subgroups.

The statistical significance level was all set at *p* < 0.05. All analyses were performed using R software ver. 4.5.1 (R Foundation for Statistical Computing, Vienna, Austria).

## Results

### Item loadings and factor variance explained

The EFA factor loadings for the Healthy Lifestyle and Behavioral components were within acceptable ranges: sleep disturbance (5 items) 0.40–0.85, physical inactivity (3 items) 0.40–0.89, suboptimal nutrition (10 items) 0.45–0.68, and substance use (8 items) 0.55–0.98. Similar ranges were observed for IRT discrimination and CFA loadings (see Supplementary Table 4).

After removing low-contributing items, the variance explained by each component increased: sleep disturbance from 38.1% to 50.6% (4-item model), physical inactivity from 20.6% to 55.0%, suboptimal nutrition from 16.2% to 54.4%, and substance use from 25.0% to 65.6%.

### Fit indices and internal consistency reliability

The developed scale met all fit index criteria, indicating acceptable model fit. Among the Healthy Lifestyle and Behavioral components, the fit indices ranged with CFI from 0.94 to 0.98, TLI from 0.93 to 0.97, RMSEA from 0.05 to 0.07, and SRMR from 0.05 to 0.07. Detailed results are shown in Supplementary Table 5.

Also, Cronbach’s alpha indicated good reliability for the sleep disturbance component (α = 0.71) and physical inactivity (α = 0.70), strong reliability for suboptimal nutrition (α = 0.82), and excellent reliability for substance use (α = 0.84).

### Risk association between HLBS and MetS

Our data has median follow-up time of 4 years (interquartile range, IQR: 2 to 8 years). Among participants who developed MetS, the median time from baseline to diagnosis was 4 years (IQR: 2 to 7 years). Baseline characteristics of the 201,123 study participants are presented in Table 1. Individuals with a low HLBS were more likely to be adults aged 20–59 years, male, hold a bachelor’s degree, and reported higher annual household income (quartile 3: 410,000–800,000 NTD; quartile 4: 810,000–1,200,000 NTD). Furthermore, moderate to low HLBS was more common among participants who developed MetS, ranging from 14.6% to 17.7%. Additionally, HLBS components including sleep disturbance, physical inactivity, suboptimal nutrition, and substance use showed significant differences across participants with varying scores.

**Table 1:** Baseline characteristics of study population

Variables	Total *N* = 201,123 (%)	Healthy lifestyle and behavior scale (HLBS)			*p*-value
		Low *n* = 69,462 (34.5)	Moderate *n* = 64,479 (32.1)	High *n* = 67,160 (33.4)	
Age					
< 20 years	4,686 (2.3)	1,158 (1.7)	2,032 (3.2)	1,496 (2.3)	< 0.001
20–39 years	106,569 (53.0)	42,157 (60.7)	34,076 (52.9)	30,336 (45.2)	
40–59 years	73,100 (36.4)	22,517 (32.4)	23,104 (35.8)	27,479 (40.9)	
≥ 60 years	16,768 (8.3)	3,630 (5.2)	5,267 (8.2)	7,849 (11.7)	
Sex					
Male	95,060 (47.3)	42,921 (61.8)	28,447 (44.1)	23,692 (35.3)	< 0.001
Female	106,063 (52.7)	26,554 (38.2)	36,041 (55.9)	43,468 (64.7)	
Education					
No formal education	2,451 (1.3)	369 (0.5)	733 (1.2)	1,349 (2.3)	< 0.001
Elementary school	12,728 (6.7)	2,917 (4.3)	4,069 (6.4)	5,742 (9.7)	
Junior high school	8,693 (4.6)	2,663 (3.9)	2,898 (4.6)	3,132 (5.3)	
High school or vocational school	36,463 (19.1)	13,471 (19.8)	11,684 (18.5)	11,308 (19.1)	
Associate degree	40,424 (21.2)	14,720 (21.6)	13,991 (22.1)	11,713 (19.8)	
Bachelor’s degree	63,754 (33.5)	23,879 (35.1)	20,881 (33.0)	18,994 (32.1)	
Graduate school or higher	26,039 (13.7)	10,022 (14.7)	9,000 (14.2)	7,017 (11.8)	
Household income					
Quartile 1	15,239 (12.8)	5,070 (9.5)	5,141 (12.3)	5,028 (21.2)	< 0.001
Quartile 2	12,961 (10.9)	5,468 (10.2)	4,510 (10.8)	2,983 (12.6)	
Quartile 3	31,141 (26.1)	14,540 (27.1)	10,847 (25.9)	5,754 (24.2)	
Quartile 4	30,345 (25.5)	14,299 (26.7)	10,852 (25.9)	5,194 (21.9)	
Quartile 5	13,003 (10.9)	6,102 (11.4)	4,758 (11.4)	2,143 (9.0)	
Quartile 6	7,363 (6.2)	3,527 (6.6)	2,631 (6.3)	1,205 (5.1)	
Quartile 7	9,186 (7.7)	4,574 (8.5)	3,163 (7.8)	1,449 (6.1)	
HLBS components					
Sleep disturbance*	0.2 (0.6)	0.2 (0.6)	0.1 (0.5)	0.3 (0.7)	< 0.001
Physical inactivity*	2.4 (3.4)	4.6 (3.9)	2.19 (3.1)	0.4 (1.0)	< 0.001
Suboptimal nutrition*	6.8 (4.7)	10.5 (4.2)	7.0 (3.3)	2.7 (2.5)	< 0.001
Substance used*	2.0 (4.5)	5.0 (6.3)	1.0 (2.3)	0.1 (0.6)	< 0.001
MetS					
No	168,937 (84.0)	57,171 (82.2)	55,062 (85.4)	56,704 (84.4)	< 0.001
Yes	32,186 (16.0)	12,304 (17.7)	9,426 (14.6)	10,456 (15.5)	

We have reported the data with categorical variables in number and percentage (%) and *continuous variables in mean and standard deviation

The number of samples included in this table was determined from the longitudinal study, based on participants’ baseline information at cohort entry

Annual household income was categorized into quartile 1 (below 400,000 NTD), quartile 2 (410,000–800,000 NTD), quartile 3 (410,000–800,000 NTD), quartile 4 (810,000–1,200,000 NTD), quartile 5 (1,210,000–1,600,000 NTD), quartile 6 (1,610,000–2,000,000 NTD), and quartile 7 (above 2,010,000 NTD)

Table 2 shows the association between HLBS and MetS development. After adjusting for covariates, moderate (RR = 1.129, 95% CI = 1.096–1.163) and low adherence (RR = 1.462, 95% CI = 1.417–1.509) to healthy lifestyle and behavior were significantly associated with MetS, compared to high adherence, with a significant p-trend indicating lower HLBS corresponded to higher MetS development (β = 0.200; *p* < 0.001). Furthermore, individual HLBS components, including sleep disturbance (RR = 1.139, 95% CI = 1.121–1.157), physical inactivity (RR = 1.043, 95% CI = 1.040–1.046), suboptimal nutrition (RR = 1.024, 95% CI = 1.021–1.028), and substance use (RR = 1.014, 95% CI = 1.011–1.018), were significantly associated with MetS risk. After adding the interaction term, a modest effect modification by baseline metabolic status was observed (*p* < 0.001), with progressively stronger associations between lower HLBS and MetS components across increasing baseline burden (1, 2, and ≥ 3 baseline components), with the highest risk observed in the ≥ 3 group.

**Table 2:** Adjusted association between healthy lifestyle and behaviors and the risk of MetS development

Variables	RR	95% CI		*p*-value
		Lower	Upper	
HLBS^				
High	1			
Moderate	1.129	1.096	1.163	< 0.001
Low	1.462	1.417	1.509	< 0.001
HLBS components				
Sleep disturbance	1.139	1.121	1.157	< 0.001
Physical inactivity	1.043	1.040	1.046	< 0.001
Suboptimal nutrition	1.024	1.021	1.028	< 0.001
Substance used	1.014	1.011	1.018	< 0.001

For the HLBS analysis, we adjusted for age, sex, education, household annual income, and calendar year. For each HLBS component, we additionally adjusted for sleep quality, physical activity, nutritional intake, and substance use, excluding the component that was being analyzed in the model

The interaction terms (HLBS × baseline) indicated increasing risk with metabolic burden in the ≥ 3 components group 1.003 (moderate HLBS) and 1.019 (low HLBS), in the 2-component group 1.005 and 1.006, and in the 1-component group 1.002 and 1.003

*HLBS* health lifestyle and behavioral scale, *MetS* metabolic syndrome, *RR* risk ratios, *CI* confidence interval

^*p*-values trend < 0.005

Further, we performed a sensitivity analysis using the modified healthy lifestyle behavior items. Detailed item descriptions are presented in Supplementary Tables 6, and the analysis yielded results consistent with the main findings, provided in Supplementary Table 7.

We stratified the analysis by age groups in Table 3. A significant linear association between HLBS and MetS development was observed across all age groups. Low adherence was significantly associated with MetS in adolescents (< 20 years) and adults (20–39 years), while both low and moderate adherence increased risk in adults 40–59 years and the elderly. For HLBS components, young adolescents were more likely to develop MetS when exposed to suboptimal nutrition. Adults were at higher risk due to all unhealthy lifestyle and behavioral components, while the elderly showed a similar pattern, except for substance use.

**Table 3:** Adjusted association between healthy lifestyle and behaviors and the risk of MetS development, stratified by age

Variables	< 20 years		20–39 years			40–59 years			≥ 60 years		
	RR	95% CI	RR		95% CI	RR		95% CI	RR		95% CI
HLBS^											
High	1		1			1			1		
Moderate	1.15	0.77–1.52	1.07		0.98–1.15	1.21*		1.16–1.27	1.17*		1.10–1.23
Low	1.44*	1.30–1.55	1.46*		1.35–1.58	1.48*		1.42–1.55	1.43*		1.34–1.53
HLBS components											
Sleep disturbance	1.12	0.66–1.91	1.15*		1.11–1.19	1.17*		1.15–1.20	1.16*		1.12–1.20
Physical inactivity	0.96	0.85–1.08	1.05*		1.04–1.06	1.04*		1.03–1.05	1.04*		1.03–1.05
Suboptimal nutrition	1.07*	1.01–1.13	1.01*		1.00–1.02	1.01*		1.00–1.02	1.01*		1.00–1.02
Substance used	1.00	0.86–1.16	1.02*		1.01–1.03	1.01*		1.00–1.02	1.00		0.99–1.01

For the HLBS analysis, we adjusted for sex, education, household annual income, and calendar year. For each HLBS component, we additionally adjusted for sleep quality, physical activity, nutritional intake, and substance use, excluding the component that was being analyzed in the model

*HLBS* health lifestyle and behavioral scale, *MetS* metabolic syndrome, *RR* risk ratios, *CI* confidence interval

*Statistical significance level set at *p* < 0.05

^*p*-values trend < 0.005 for all age groups

Table 4 shows sex-stratified analyses. A significant linear association between HLBS and MetS was observed in both sexes, with low to moderate adherence increasing MetS risk. Most HLBS components were associated with MetS in both males and females, except substance use, which was not linked to MetS development among females.

**Table 4:** Adjusted association between healthy lifestyle and behaviors and the risk of MetS development, stratified by sex

Components	Male		Female	
	RR	95% CI	RR	95% CI
HLBS^				
High	1		1	
Moderate	1.14*	1.08–1.18	1.13*	1.09–1.18
Low	1.53*	1.46–1.59	1.41*	1.35–1.48
HLBS components				
Sleep disturbance	1.12*	1.09–1.14	1.16*	1.14–1.19
Physical inactivity	1.05*	1.04–1.06	1.03*	1.02–1.04
Suboptimal nutrition	1.02*	1.01–1.03	1.01*	1.00–1.02
Substance used	1.02*	1.01–1.03	1.00	0.98–1.01

For the HLBS analysis, we adjusted for age, education, household annual income, and calendar year. For each HLBS component, we additionally adjusted for sleep quality, physical activity, nutritional intake, and substance use, excluding the component that was being analyzed in the model

*HLBS* health lifestyle and behavioral scale, *MetS* metabolic syndrome, *RR * risk ratos, *CI* confidence interval

*Statistical significance level set at *p* < 0.05

^*p*-values trend < 0.005 for sex

## Discussion

Our study found that individuals with moderate to low adherence to healthy lifestyle behaviors had a 12.9% to 46.2% increased risk of developing MetS, consistent with previous findings [11, 12]. Even after stratifying by age and sex, the linear association between low adherence to healthy lifestyle behaviors and increased risk of MetS remained evident across all age groups and both sexes, but was most pronounced among adults and the elderly. Poor lifestyle factors such as sleep disturbance, physical inactivity, suboptimal nutrition, and substance use may strongly influence MetS development, as supported by previous studies [9, 10, 12].

With the developed HLBS, we can predict health outcomes such as the risk of developing MetS. While previous scales informed the HLBS, they were often limited in scope [13], addressing only parts of the framework such as sleep, stress, physical activity, nutrition, social connectedness, or substance use [11, 12, 17]. To capture the major health components, we identified and validated most HLBS domains using a Taiwanese health dataset. Furthermore, validated instruments such as the RU SATED sleep questionnaire, the Mini EAT nutrition tool, and the Muscle Strengthening Exercise Questionnaire (MSEQ) for physical activity were mainly developed in Western settings and later adapted for HLBS development [13]. Moreover, cultural, social, and environmental factors strongly influence population behaviors related to health components [30]. For instance, differences in sleep-related behaviors and reported sleep quality have been observed in some Asian populations, which may be influenced by urban living conditions, occupational or academic demands, and lifestyle-related factors. In Western populations, some studies have reported differences in subjective sleep quality, although sleep disorders such as sleep apnea are more frequently diagnosed [30]. Regarding diet, traditional many Asian diets are largely plant-based, emphasizing rice, vegetables, soy, fish, and tea, but increasing Westernization has led to greater consumption of processed foods, sugars, and fats in many Asian countries [31]. Similarly, physical activity patterns may differ across settings, with some Asian populations engaging more in incidental activity such as walking or cycling, whereas leisure-time or structured exercise such as gym-based training and high-intensity activities is more commonly reported in Western populations [32]. Given these regional variations in health behavior, the HLBS developed in this study provides a more culturally and contextually appropriate measure of healthy lifestyle behaviors in some Asian populations. Using items identified from the Taiwanese health dataset allows the scale to capture locally relevant behaviors, enhancing the validity and applicability of health assessments. This development also has important implications for public health research and practice by enabling more precise identification of behavioral risk factors, informing culturally tailored interventions, and supporting cross-population comparisons that recognize regional lifestyle differences. Moreover, future studies are needed across Asian regions, as it comprises highly heterogeneous cultural, environmental, and behavioral patterns, which may in turn influence health-related lifestyle behaviors.

Using the HLBS, we observed an increased MetS development among individuals with low healthy lifestyle adherence. Each component, including sleep disturbance, physical inactivity, suboptimal nutrition, and substance use, independently contributed, and their interaction further compounded adverse effects on metabolic health [11]. This supports evidence that lifestyle behaviors influence MetS through interconnected physiological and behavioral pathways [11, 33–35]. For example, disrupted sleep can dysregulate appetite-related hormones, reducing leptin and increasing ghrelin, which heightens cravings for high-calorie foods and late-night snacking, leading to overeating, weight gain, and further sleep disruption [33]. Sleep disturbance also impairs glucose metabolism, increases cortisol levels, and triggers systemic inflammation, all contributing to insulin resistance and elevated blood pressure [33, 34]. Fatigue from poor sleep can reduce physical activity, whereas regular exercise can improve sleep quality [34]. Physical inactivity often co-occurs with poor dietary habits, amplifying weight gain and insulin resistance [35]. Suboptimal nutrition, including high intake of processed foods, sugars, and saturated fats, raises triglycerides, reduce HDL-C, and promote central adiposity [11, 36]. Substance use such as alcohol and tobacco, worsens metabolic risk by increasing oxidative stress, impairing liver function, and disrupting lipid and glucose metabolism [11, 37]. These behaviors interact with poor sleep and physical inactivity, amplifying adverse effects [36, 37]. Overall, the combined impact of multiple unhealthy behaviors exceeds the sum of individual effects, highlighting the need to address multiple lifestyle factors simultaneously to reduce MetS development.

After stratifying by age, a consistent linear trend was observed between healthy lifestyle behaviors and MetS risk development across all age-groups. Individuals with healthier lifestyle had lower MetS development regardless of age. However, the association was stronger among older adults and the elderly, indicating that poor lifestyle habits have a more pronounced impact on MetS development later in life. With age, physiological changes such as reduced insulin sensitivity, slower metabolism, and increased abdominal fat make individuals more vulnerable to metabolic disturbances [38]. Additionally, older adults may have prolonged exposure to unhealthy behaviors, such as poor diet or physical inactivity, which can exacerbate metabolic dysfunction [39]. The natural decline in physiological resilience and hormonal balance with aging also reduces the body’s ability to compensate for unhealthy habits [38, 39]. Age-related comorbidities, such as hypertension and diabetes, can further interact with lifestyle factors, amplifying MetS development [38, 39]. These findings suggest that maintaining healthy lifestyle behaviors becomes increasingly important with age to mitigate the heightened susceptibility to metabolic disorders. Meanwhile, when examining individual components by age, most were significantly associated with MetS development across all groups, though only suboptimal nutrition was significant among adolescents. Adolescents generally have higher metabolic resilience, which helps offset the immediate impact of behaviors like physical inactivity or irregular sleep [40, 41]. Adolescents also have shorter cumulative exposure to unhealthy behaviors than older adults, so factors like inactivity or poor sleep may not yet impact MetS development [40, 41]. In contrast, nutrition directly influences energy balance, lipid profiles, and insulin sensitivity, meaning that poor dietary habits such as high sugar, processed food, low fruit and vegetable consumption can cause early metabolic changes [42]. Additionally, lifestyle risks such as alcohol consumption or smoking are less prevalent in adolescents, making nutrition a dominant determinant of early metabolic risk [40, 41]. These factors explain why suboptimal nutrition stands out as the only component significantly associated with MetS among adolescents.

Stratified analysis by sex showed a linear trend between unhealthy lifestyle behavior and the development of MetS. While substance use was not associated with MetS in females, other components such as sleep disturbance, physical inactivity, and suboptimal nutrition were significantly linked to MetS in both sexes. The lack of association in females may reflect their lower prevalence and intensity of substance use compared to men [43]. In Taiwan, although alcohol consumption among women is rising, occasional to heavy drinking remains more common in men [43]. Meanwhile, health-related behaviors such as sleep disturbance, physical inactivity, and suboptimal nutrition are more universally experienced across both sexes and directly affect key metabolic processes, including insulin sensitivity, lipid metabolism, and energy balance [11]. These behaviors exert both immediate and cumulative effects on metabolic health, which may explain their consistent association with MetS in males and females, despite sex-specific lifestyle differences.

Our study has several strengths. Utilizing the MJ Health Management Institution dataset, which spans 24 years, allowed for a more accurate assessment of the temporal development of MetS. The dataset’s detailed and repeated measurements of lifestyle factors and MetS-related health indicators enhance the reliability of our findings. Leveraging a large cohort-based source, we were able to exclude baseline MetS cases while maintaining a sufficient sample size to reliably examine disease progression and conduct stratified analyses. We developed an overall HLBS incorporating most of the recommended components, including sleep disturbance, physical inactivity, and suboptimal nutrition. In the main analysis, we were also able to adjust for demographic factors and lifestyle behaviors. For the developed scale, we utilized IRT, as recommended [13], to assess item discrimination and difficulty, improving on classical test theory’s limitations in evaluating item-level properties.

Moreover, our study has certain limitations. Although most HLBS components were included, we were unable to incorporate other important health factors such as stress and social connections, which are also key elements of a healthy lifestyle and may influence the risk of developing health outcomes such as MetS. The use of an open cohort design may introduce variability in follow-up time, potential attrition bias, and cohort heterogeneity, as participants enter and leave the study at different periods. However, we assessed intermittent missingness and performed CFA models estimated using FIML. We also excluded participants lacking information on healthy lifestyle behaviors or MetS follow-up, considering only those with sufficient data. Although FIML assumes data are missing at random, part of the missingness in this cohort is missing-by-design due to changes in questionnaire versions across study waves. While we conducted a sensitivity analysis restricted to the 2017–2023 period, during which most questionnaire items had been updated to improve consistency and comparability, cross-wave measurement invariance testing was limited within the harmonized dataset and thus evidence supporting measurement equivalence across questionnaire eras remains potentially constrained. Further, this study may have limited representativeness, as participants were drawn only from individuals who visited hospitals or from organizations that required employees to undergo clinical examinations. Future studies should consider using population-based data from multiple catchment areas to enhance the generalizability of the findings.

## Conclusions

The developed HLBS provides a culturally relevant tool for assessing healthy lifestyle behaviors, improving risk prediction and guiding interventions. Low to moderate adherence significantly increases MetS development, especially in adults and the elderly. Key behaviors such as sleep disturbance, physical inactivity, suboptimal nutrition, and substance use independently and collectively amplify risk, highlighting the multifactorial impact of lifestyle. These findings support public health policies promoting comprehensive healthy behaviors to prevent and manage MetS.

## Supplementary Information

Supplementary Material 1.

## Data Availability

The data supporting the findings of this study are available from the MJ Health Research Foundation. However, access to these data is restricted, as they were provided for use under specific approval for the current study and are therefore not publicly available. Researchers interested in applying for access to the dataset may contact the corresponding author, Dr. Ta-Chien Chan (E-mail: tachien@sinica.edu.tw) for further information regarding the application process.

## Abbreviations

CI: 95% Confidence intervals
CFA: Confirmatory factor analysis
CFI: Comparative fit index
EFA: Exploratory factor analysis
FIML: Full information maximum likelihood
HLBS: Healthy lifestyle and behavioral scale
HDL-C: High-density lipoprotein cholesterol
IQR: Interquartile range
IRT: Item response theory
MJ: Mei Jau Health Management
RR: Risk ratios
RMSEA: Root means square error of approximation
SRMR: Standardized root means square residual
TLI: Tucker-Lewis Index
