# Supplementary material for: Development and validation of a healthy lifestyle and behavioral score for predicting metabolic syndrome: evidence from a 24-year longitudinal health check-up cohort in Taiwan
Source: Int J Behav Nutr Phys Act. 2026 Jun 18;23:76. doi: 10.1186/s12966-026-01940-x (PMC13445918; doi:10.1186/s12966-026-01940-x)
Supplement: Supplementary file 1 [file 12966_2026_1940_MOESM1_ESM.docx]

**MetS**
Without MetS: n= 168,937 (84.0%)
With MetS: n= 32,186 (16.0%)

**MetS**
Without MetS: n= 438,324 (83.8%)
With MetS: n= 84,858 (16.2%)

Excluded participants with ≥3 missing MetS measurements, only one health check visit in the cohort, or MetS at baseline (n=322,239)

**MetS measurements from the Health Dataset
2000 to 2023**

**Lower high-density lipoprotein cholesterol
(HDL-C)**
(n= 483,273)

Men (<40 mg/dL),
where mean: 44.7 (sd:9.7)

Women (<50 mg/dL),
where mean: 52.3 (sd:13.1)

**Hypertriglyceridemia**
(n= 523,342)

Triglyceride levels ≥150 mg/dL,
where mean: 234.8 (sd:155.0)

Long-term use of drugs for hyperlipidemia, where n= 8,116 (1.5%)

**Hyperglycemia**
(n= 523,323)

Fasting plasma glucose (FPG) ≥100 mg/dL, where mean: 114.2 (sd:30.1)

Antihyperglycemic medication users, where n= 13,457 (2.6%)

**Hypertension**
(n= 523,356)

Systolic BP ≥130 mmHg (left arm), where mean: 141.5 (sd:16.0)
Systolic BP ≥130 mmHg (right arm), where mean: 138.9 (sd:14.5)

Diastolic BP ≥85 mmHg (left arm), where mean: 84.4 (sd:10.9)
Diastolic BP ≥85 mmHg (right arm), where mean: 83.6 (sd:10.4)

Antihypertensive drug users,
where n= 7,434 (3.7%)

**Abdominal obesity**
(n= 513,618)

Men (≥90 cm),
where mean: 96.7 (sd:5.9)

Women (≥80 cm),
where mean: 87.2 (sd:6.2)

**Without abdominal obesity**
n= 428,488 (83.4%)

**With abdominal obesity**
n= 85,130 (16.6%)

**Without hypertension**
n= 377,036 (72.0%)

**With hypertension**
n= 146,320 (28.8%)

**Without hyperglycemia**
n= 333,551 (63.7%)

**With hyperglycemia**
n= 189,772 (36.2%)

**Without hypertriglyceridemia**
n= 416,090 (79.5%)

**With hypertriglyceridemia**
n= 107,252 (20.4%)

**Without lower HDL-C**
n= 398,754 (82.5%)

**With lower HDL-C**
n= 84,519 (17.4%)

*MetS, metabolic syndrome; BP, blood pressure; sd, standard deviation*
**Supplementary Figure 1**. Measurement used to identify patients with incidence of metabolic syndrome in the population.

**Supplementary Table 1.** Sample selection and annual incidence rate of MetS per year in an open cohort.

| Year | Number of patient visits | | | | Number of study samples | | | | | |
| --- | --- | --- | --- | --- | --- | --- | --- | --- | --- | --- |
|  | Total no. of visits in the baseline cohort | Total no. of visits excluding missing MetS measurements | Total no. of patients with ≥2 health check visits | Total no. of visits excluding baseline incidence | Total no. of samples in the baseline cohort | Total no. of samples without missing Mets measurements | Total no. of samples with ≥ 2 health check visits | Total no. of study samples excluding baseline incidence | Incidence cases (MetS cases in a particular year | Population at -risk |
| 2000 | 79,059 | 79,059 | 48,700 | 41,101 | 43,656 | 43,660 | 14,913 | 11,139 | 54 | 9,316 |
| 2001 | 71,198 | 71,198 | 49,286 | 41,726 | 35,216 | 35,447 | 15,670 | 11,323 | 1,509 | 9,588 |
| 2002 | 73,417 | 73,417 | 52,262 | 44,630 | 34,968 | 35,012 | 16,205 | 11,602 | 1,691 | 9,814 |
| 2003 | 68,093 | 68,093 | 50,959 | 43,702 | 29,977 | 30,005 | 15,368 | 10,800 | 1,816 | 9,117 |
| 2004 | 74,846 | 74,846 | 58,360 | 50,033 | 30,873 | 30,867 | 16,065 | 12,150 | 1,740 | 10,214 |
| 2005 | 78,688 | 78,687 | 60,919 | 52,573 | 32,839 | 32,605 | 14,040 | 12,890 | 1,744 | 10,909 |
| 2006 | 81,608 | 81,608 | 64,755 | 55,671 | 32,457 | 32,599 | 14,966 | 13,542 | 2,433 | 11,325 |
| 2007 | 80,790 | 80,790 | 63,565 | 54,539 | 32,292 | 32,379 | 14,796 | 12,572 | 2,815 | 10,401 |
| 2008 | 80,629 | 80,583 | 65,338 | 56,384 | 30,568 | 30,648 | 14,320 | 13,380 | 2,235 | 11,136 |
| 2009 | 76,088 | 76,065 | 64,454 | 55,531 | 26,616 | 26,574 | 13,171 | 12,791 | 2,152 | 10,699 |
| 2010 | 74,742 | 74,734 | 60,592 | 52,597 | 28,715 | 28,538 | 13,138 | 12,391 | 1,895 | 10,481 |
| 2011 | 67,303 | 67,287 | 54,793 | 47,694 | 25,337 | 25,250 | 12,142 | 11,147 | 1,883 | 9,458 |
| 2012 | 69,506 | 69,506 | 53,589 | 46,913 | 28,941 | 28,726 | 11,867 | 11,343 | 1,793 | 9,635 |
| 2013 | 58,899 | 58,899 | 44,969 | 39,412 | 24,499 | 24,736 | 9,537 | 9,276 | 1,632 | 7,774 |
| 2014 | 28,195 | 28,192 | 24,488 | 21,390 | 8,749 | 8,799 | 4,759 | 4,524 | 923 | 3,692 |
| 2015 | 30,418 | 30,418 | 24,845 | 21,760 | 10,953 | 10,972 | 5,154 | 4,828 | 995 | 3,988 |
| 2016 | 26,414 | 26,410 | 21,571 | 18,991 | 9,557 | 9,535 | 4,420 | 4,185 | 736 | 3,472 |
| 2017 | 22,718 | 22,718 | 17,826 | 15,622 | 8,914 | 8,932 | 3,893 | 3,448 | 732 | 2,833 |
| 2018 | 24,471 | 24,462 | 19,836 | 17,394 | 9,173 | 9,072 | 4,187 | 3,858 | 764 | 3,243 |
| 2019 | 24,075 | 24,058 | 19,879 | 17,474 | 9,032 | 8,970 | 4,667 | 4,293 | 799 | 3,588 |
| 2020 | 20,072 | 20,051 | 15,922 | 14,083 | 7,891 | 7,913 | 3,766 | 3,372 | 691 | 2,812 |
| 2021 | 14,504 | 14,504 | 10,342 | 9,158 | 6,861 | 6,881 | 2,629 | 2,397 | 457 | 2,062 |
| 2022 | 14,393 | 14,393 | 9,280 | 8,212 | 7,728 | 7,665 | 2,679 | 2,272 | 387 | 2,005 |
| 2023 | 12,739 | 12,739 | 6,997 | 6,272 | 7,625 | 7,597 | 1,840 | 1,600 | 310 | 1,375 |
| Total | 1,252,865 | 1,252,717 | 963,527 | 832,862 | 523,437 | 523,382 | 234,192 | 201,123 | 32,186 | 168,937 |

MetS, metabolic syndrome.

**Supplementary Table 2.** Full set of items tested for the development and psychometric evaluation of the healthy lifestyle and behavioral scale using the MJ Health Database.

| No. | Variable description | Response type | No. of categories | Coding | IRT model | Men  Mean (SD) | Women  Mean (SD) | Total Mean (SD) | p-value |
| --- | --- | --- | --- | --- | --- | --- | --- | --- | --- |
| Sleep disturbance | | | | | | | | | |
| 1 | How many hours of sleep do you get every day? | Ordinal | 4 | 0=0–4 hrs.; 1=4–6 hrs.; 2=6–8 hrs.; 3=> 8 hrs. | GRM | 1.85 (0.52) | 1.85 (0.55) | 1.85 (0.53) | <0.001 |
| 2 | How has the quality of your sleep been in the past month? | Ordinal | 5 | 0=Difficult falling asleep; 1=Able to fall asleep but easily awakened; 2=Frequent dreams; 3=Needing sleeping pills or sedatives; 4=Deep sleep | GRM | 2.56 (1.50) | 2.13 (1.45) | 2.34 (1.49) | <0.001 |
| 3 | Have you had difficulty falling asleep in the past month? | Dichotomous | 2 | 0=No; 1=Yes | 2PL | 0.13 (0.34) | 0.19 (0.39) | 0.16 (0.36) | <0.001 |
| 4 | Have you taken sleeping pills or sedatives to help you fall asleep in the past month? | Dichotomous | 2 | 0=No; 1=Yes | 2PL | 0.02 (0.15) | 0.04 (0.20) | 0.03 (0.18) | <0.001 |
| 5 | During the past month, could you fall asleep but were easily awakened? | Dichotomous | 2 | 0=No; 1=Yes | 2PL | 0.28 (0.44) | 0.38 (0.48) | 0.33 (0.47) | <0.001 |
| 6 | In the past month, have you had frequent dreams during sleep? | Dichotomous | 2 | 0=No; 1=Yes | 2PL | 0.18 (0.39) | 0.27 (0.44) | 0.23 (0.42) | <0.001 |
| 7 | In the past month, would you say you rarely experienced deep sleep? | Dichotomous | 2 | 0=No; 1=Yes | 2PL | 0.45 (0.49) | 0.33 (0.47) | 0.39 (0.48) | <0.001 |
| 8 | In the past month, did you often feel tired upon waking? | Dichotomous | 2 | 0=No; 1=Yes | 2PL | 0.35 (0.47) | 0.37 (0.48) | 0.36 (0.48) | <0.001 |
| Physical inactivity | | | | | | | | | |
| 1 | How would you describe the intensity of your daily work activities? | Ordinal | 4 | 0=Physically active most of the time; 1= Mostly standing and walking; 2= Frequently sitting and walking; 3= Sitting most if the time | GRM | 2.44 (0.80) | 2.61 (0.64) | 2.53 (0.72) | <0.001 |
| 2 | What type of exercise do you do most often | Ordinal | 5 | 0=Vigorous exercise (gym lifting, running, skipping etc.); 1= Heavy exercise (e.g., jogging, climbing, etc.); 2= Moderate exercise (e.g. playing sports, swimming, etc.); 3= Light exercise (e.g. brisk walking, cycling, etc.); 4=No exercise | GRM | 2.54 (0.76) | 2.90 (0.50) | 2.72 (0.67) | <0.001 |
| 3 | What is your usual scheduled weekly time for exercise? | Ordinal | 5 | 0=7 hrs. or more; 1=5-6 hrs.; 2=3-4 hrs.; 3=1-2 hrs.; 4= None or less than 1 hr. | GRM | 2.82 (1.20) | 3.10 (1.51) | 2.97 (1.16) | <0.001 |
| 4 | How many times have you exercised regularly in the past two weeks? | Ordinal | 5 | 0=2-3 time a day; 1= Once a day; 2= Once every 2-3 days; 3= Once a week; 4= No exercise or only once in a long time | GRM | 2.51 (1.12) | 2.48 (1.20) | 2.49 (1.16) | <0.001 |
| 5 | How much time do you usually spend exercising per session? | Ordinal | 4 | 0= More than 2 hrs.; 1=1-2 hrs.; 2=0.5-1 hr.; 3=Less than 0.5 hrs. | GRM | 1.82 (0.90) | 2.01 (0.82) | 1.92 (0.87) | <0.001 |
| 6 | Are there any reasons why you cannot exercise? (e.g., health issues) | Dichotomous | 2 | 0=No; 1=Yes | 2PL | 0.04 (0.21) | 0.06 (0.24) | 0.05 (0.23) | <0.001 |
| Suboptimal nutrition | | | | | | | | | |
| 1 | Do you eat at regular times and in regular amounts? | Dichotomous | 2 | 0=No; 1=Yes | 2PL | 0.67 (0.46) | 0.66 (0.47) | 0.66 (0.47) | <0.001 |
| 2 | At what time do you usually have your final meal each day? | Ordinal | 6 | 0=Before 17:00; 1=17:00-18:00; 2=18:00-19:00; 3=19:00-20:00; 4=20:00-21:00; 5=After 21:00 | GRM | 2.98 (1.13) | 2.86 (1.13) | 2.92 (1.13) | <0.001 |
| 3 | Do you usually take any nutritional supplements? | Dichotomous | 2 | 0=No; 1=Yes | 2PL | 0.47 (0.49) | 0.36 (0.48) | 0.42 (0.49) | <0.001 |
| 4 | Are you a full-time vegetarian? | Dichotomous | 2 | 0=No; 1=Yes | 2PL | 0.02 (0.14) | 0.03 (0.18) | 0.03 (0.17) | <0.001 |
| 5 | How frequently do you consume fried or pan-fried foods? (1 serving is about half a bowl). | Ordinal | 5 | 0=No or less than 1 serving per week; 1=1-3 servings per week; 2=4-6 servings per week; 3=1 serving per day; 4=2 or more servings per day | GRM | 0.96 (0.80) | 0.82 (0.78) | 0.89 (0.79) | <0.001 |
| 6 | How frequently do you consume fried, deep-fried, deep-fried, grilled, or smoked foods? | Ordinal | 4 | 0=No or less than 1 serving per week; 1=2-3 servings per week; 2=4-5 servings per week; 3=6 serving per day or everyday | GRM | 0.84 (0.73) | 0.71 (0.70) | 0.77 (0.72) | <0.001 |
| 7 | How much non-dark green vegetables do you usually eat (e.g., cabbage, bok choy, cucumber, radish, etc.) | Ordinal | 5 | 0=No or less than half a bowl a day; 1= Half a bowl to less than 1 bowl a day; 2=1 bowl to less than 1.5 bowl a day; 3=1.5 bowls to less than 2 bowls a day; 4=2 bowls or more a day | GRM | 1.29 (0.79) | 1.26 (0.82) | 1.28 (0.80) | <0.001 |
| 8 | How many servings of vegetables do you usually consume in a day? | Ordinal | 5 | 0=No or less than half a bowl a day; 1=Half a bowl to less than 1 bowl a day; 2=1 bowl to less than 1.5 bowl a day; 3=1.5 bowls to less than 2 bowls a day; 4=2 bowls or more a day | GRM | 1.48 (0.93) | 1.49 (0.99) | 1.48 (0.96) | <0.001 |
| 9 | How many servings of vegetables do you consume that are stir-fried with oil or prepared with salad dressing? | Ordinal | 5 | 0=No or less than half a bowl a day; 1=Half a bowl to less than 1 bowl a day; 2=1 bowl to less than 1.5 bowl a day; 3=1.5 bowls to less than 2 bowls a day; 4=2 bowls or more a day | GRM | 0.84 (0.73) | 0.87 (0.79) | 0.86 (0.77) | <0.001 |
| 10 | How many servings of fruit do you usually consume? (1 serving is about half medium of fruits such as apple, grapefruit, guava, etc.; 1 fruit such as orange, tangerine, kiwi, etc.; half a pound of fruits such as papaya, watermelon, etc.; 5 fruits such as lychees, etc.; 12 fruits such as grapes, longans, etc.) | Ordinal | 5 | 0=No or less than 1 serving per day; 1=1-2 servings per day; 2=2-3 servings per day; 3=3-4 servings per day; 4=4 or more servings per day | GRM | 0.86 (0.72) | 0.92 (0.71) | 0.89 (0.71) | <0.001 |
| 11 | Do you usually consume at least 2 servings of fruit per day? (1 serving is about 1 first size fruits such as apple, grapefruit, guava, orange, or tangerine, etc.; 1 palm-size of fruits such as papaya, watermelon, etc.; 1 handful of fruits such as lychees, grapes, etc.) | Ordinal | 4 | 0=Never; 1=Occasionally; 2=Often; 3=Always | GRM | 1.41 (0.65) | 1.49 (0.70) | 1.45 (0.68) | <0.001 |
| 12 | How many servings of rice or noodles do you usually consume? (1 serving is about 1 bowl of rice; 2 bowls of noodles or rice vermicelli; 4 thin slices of toast; one steamed bun; 2 pieces of pan-fried cakes/fried dough sticks) | Ordinal | 5 | 0=No or less than 1 serving per day; 1=1-2 servings per day; 2=2-3 servings per day; 3=3-4 servings per day; 4=4 or more servings per day | GRM | 1.57 (0.79) | 1.20 (0.66) | 1.38 (0.75) | <0.001 |
| 13 | What portion of your daily rice or noodle intake consists of who grains? (1 serving is about 4 slices of whole-wheat bread; 1 bowl of brown or multi-grain rice; 8 tablespoons of oatmeal). | Ordinal | 5 | 0=No or less than 1 serving per week; 1=1-3 servings per week; 2=4-6 servings per week; 3=1 servings per day; 4=2 or more servings per day | GRM | 0.92 (1.04) | 0.83 (1.00) | 0.87 (1.02) | <0.001 |
| 14 | How frequently do you choose germ rice, brown rice, whole-grain rice, or whole-wheat pasta? | Ordinal | 4 | 0=Never; 1=Occasionally; 2=Often; 3=Always | GRM | 1.04 (0.68) | 1.12 (0.73) | 1.08 (0.71) | <0.001 |
| 15 | What portion of your rice or noodle intake is fried or cooked with oil? (1 serving is about 1 bowl of fried rice, fried noodles, or fried rice noodles; or 2 pieces of pan-fried cakes or fried dough sticks). | Ordinal | 5 | 0=No or less than 1 serving per week; 1=1-3 servings per week; 2=4-6 servings per week; 3=1 servings per day; 4=2 or more servings per day | GRM | 0.69 (0.75) | 0.56 (0.73) | 0.62 (0.74) | <0.001 |
| 16 | How many servings of baked goods (such as sweet bread) do you usually eat? (1 serving is about 1 red bean bread, 1 crisp bread, 1 cake, or 15 sweet cookies). | Ordinal | 5 | 0=No or less than 1 serving per week; 1=1-3 servings per week; 2=4-6 servings per week; 3=1 serving per day; 4=2 or more servings per day | GRM | 1.06 (0.85) | 1.03 (0.86) | 1.05 (0.86) | <0.001 |
| 17 | How many servings of pickles, processed foods (such as ham, sausage, canned food, etc.), or salted fish do you usually eat? (1 serving is about one tablespoon of pickles, half a sausage, 2 slices of ham, or 1 tablespoon of canned meat). | Ordinal | 5 | 0=No or less than 1 serving per week; 1=1-3 servings per week; 2=4-6 servings per week; 3=1 serving per day; 4=2 or more servings per day | GRM | 0.47 (0.62) | 0.34 (0.56) | 0.40 (0.59) | <0.001 |
| 18 | How often do you eat instant noodles? (1 serving is about 1 bowl or 1 pack of instant noodles). | Ordinal | 5 | 0=No or less than 1 serving per week; 1=1-3 servings per week; 2=4-6 servings per week; 3=1 serving per day; 4=2 or more servings per day | GRM | 0.22 (0.45) | 0.16 (0.39) | 0.19 (0.42) | <0.001 |
| 19 | Do you usually dip your food in soy sauce or other salty condiments? (1 serving is about 2 teaspoons of soy sauce, ketchup, chili sauce, or black vinegar, or 1 teaspoon of salt and pepper). | Ordinal | 5 | 0=No or less than 1 serving per week; 1=1-3 servings per week; 2=4-6 servings per week; 3=1 serving per day; 4=2 or more servings per day | GRM | 0.69 (0.79) | 0.58 (0.77) | 0.63 (0.78) | <0.001 |
| 20 | How often do you dip your food in sauces or chili sauce? | Ordinal | 4 | 0=Never; 1=Occasionally; 2=Often; 3=Always | GRM | 1.28 (0.61) | 1.23 (0.64) | 1.25 (0.63) | <0.001 |
| 21 | When cooking, how often do you add extra soy sauce or other seasonings to enhance the flavor of food? | Ordinal | 4 | 0=Never; 1=Occasionally; 2=Often; 3=Always | GRM | 1.31 (0.60) | 1.33 (0.62) | 1.32 (0.61) | <0.001 |
| 22 | How often do you eat foods with jam or honey? (1 serving is about to about 2 teaspoons of jam or honey). | Ordinal | 5 | 0=No or less than 1 serving per week; 1=1-3 servings per week; 2=4-6 servings per week; 3=1 serving per day; 4=2 or more servings per day | GRM | 0.26 (0.54) | 0.24 (0.53) | 0.25 (0.53) | <0.001 |
| 23 | How often do you add extra sugar to your diet? (1 serving is about 2 teaspoons of white sugar, honey, jam, or fructose, or 3 sugar cubes). | Ordinal | 5 | 0=No or less than 1 serving per week; 1=1-3 servings per week; 2=4-6 servings per week; 3=1 serving per day; 4=2 or more servings per day | GRM | 0.40 (0.73) | 0.39 (0.72) | 0.39 (0.73) | <0.001 |
| 24 | How often do you eat sweet foods? | Ordinal | 4 | 0=No or once a week; 1=2-3 times a week; 2=4-5 times a week; 3=6 times a week or every day | GRM | 0.75 (0.75) | 0.80 (0.77) | 0.78 (0.76) | <0.001 |
| 25 | How often do you drink coffee, cocoa, tea, juice, or other beverages with added sugar (such as soda or sweetened mung bean soup)? (1 cup = 240 ml). | Ordinal | 5 | 0=No or less than 1 drink per week; 1=1-3 drinks per week; 2=4-6 drinks per week; 3=1 drink per day; 4=2 or more drinks per day | GRM | 1.38 (1.26) | 1.16 (1.20) | 1.26 (1.24) | <0.001 |
| 26 | How often do you drink fresh fruit juice? | Ordinal | 4 | 0=Never; 1=Occasionally; 2=Often; 3=Always | GRM | 1.01 (0.60) | 0.93 (0.62) | 0.97 (0.61) | <0.001 |
| 27 | How much milk do you usually drink? (1 cup = 240 ml of fresh milk, 240 ml of yogurt, or 4 level tablespoons of milk powder). | Ordinal | 5 | 0=No or less than 1 drink per week; 1=1-3 drinks per week; 2=4-6 drinks per week; 3=1 drink per day; 4=2 or more drinks per day | GRM | 0.82 (1.01) | 0.90 (1.04) | 0.86 (1.03) | <0.001 |
| 28 | How much dairy products do you usually eat? (1 serving is about 1 small cup of yogurt or 1 slice of or cheese). | Ordinal | 5 | 0=No or less than 1 serving per week; 1=1-3 servings per week; 2=4-6 servings per week; 3=1 serving per day; 4=2 or more servings per day | GRM | 0.49 (0.69) | 0.53 (0.72) | 0.51 (0.70) | <0.001 |
| 29 | How much beans and soy products do you usually eat? (1 serving is about half a box of tofu, 1 cup = 240 ml of soy milk, 2 blocks of five-spice dried tofu, or half a palm-sized piece of tofu) | Ordinal | 5 | 0=No or less than 1 serving per week; 1=1-3 servings per week; 2=4-6 servings per week; 3=1 serving per day; 4=2 or more servings per day | GRM | 1.28 (0.85) | 1.23 (0.87) | 1.25 (0.86) | <0.001 |
| 30 | How much seafood do you usually eat? (1 serving is about 1 medium-sized saury, 4 slices of sashimi, 4 tablespoons of cooked fish, 4 grass shrimp, or 16 oysters). | Ordinal | 5 | 0=No or less than 1 serving per week; 1=1-3 servings per week; 2=4-6 servings per week; 3=1 serving per day; 4=2 or more servings per day | GRM | 1.25 (0.82) | 1.20 (0.84) | 1.23 (0.83) | <0.001 |
| 31 | How many eggs do you usually eat? (1 serving is about 1 chicken or duck egg, or 5 pigeon eggs) | Ordinal | 5 | 0=No or less than 1 serving per week; 1=1-3 servings per week; 2=4-6 servings per week; 3=1 serving per day; 4=2 or more servings per day | GRM | 1.47 (0.86) | 1.27 (0.81) | 1.37 (0.84) | <0.001 |
| 32 | How much meat do you usually eat (such as pork, chicken, duck, lamb, or beef)? (1 plate is about 1 slice of pork steak [palm-sized, 1 cm thick], 1 pork shank leg, 1 hamburger patty, or 4 tablespoons of other lean meat). | Ordinal | 5 | 0=No or less than 1 serving per week; 1=1-3 servings per week; 2=4-6 servings per week; 3=1 serving per day; 4=2 or more servings per day | GRM | 2.03 (1.02) | 1.73 (1.00) | 1.88 (1.02) | <0.001 |
| 33 | How much offal do you usually eat (such as liver, kidney, heart, intestines, etc.)? (1 serving is about half a bowl of pork or chicken liver, 8 chicken hearts, or 4 tablespoons of cooked pork intestines). | Ordinal | 5 | 0=No or less than 1 serving per week; 1=1-3 servings per week; 2=4-6 servings per week; 3=1 serving per day; 4=2 or more servings per day | GRM | 0.32 (0.54) | 0.22 (0.46) | 0.27 (0.51) | <0.001 |
| Substance use | | | | | | | | | |
| 1 | Do you smoke? | Ordinal | 6 | 0=Do not smoke; 1=Do not smoke, but often inhale secondhand smoke; 2=Used to smoke, but have quit; 3=Smoke 1-3 times a week; 4=Smoke 4-5 times a week; 5=Smoke every day | GRM | 1.79 (2.16) | 0.31 (1.04) | 1.01 (1.83) | <0.001 |
| 2 | How many years have you been smoking? | Ordinal | 5 | 0= Do not smoke; 1=Less than 1 year; 2=1-3 years; 3=3-10 years; 4=More than 10 years | GRM | 3.11 (2.17) | 0.72 (1.60) | 2.16 (2.29) | <0.001 |
| 3 | How many cigarettes do you usually smoke per day? (If you have quit, please answer based on your past smoking habits). | Ordinal | 3 | 0=Less than half a pack per day; 1=Half a pack to one pack per day; 2=More than one pack per day | GRM | 0.09 (0.06) | 0.01 (0.12) | 0.03 (0.37) | <0.001 |
| 4 | On average, how many cigarettes do you smoke per day? (If you have quit, please answer based on your past experience). | Ordinal | 3 | 0=Less than half a pack; 1=Half a pack to one pack; 2=More than one pack | GRM | 1.10 (1.44) | 0.11 (0.51) | 0.58 (1.17) | <0.001 |
| 5 | Do you drink alcohol? | Ordinal | 5 | 0=Do not drink or drink less than once a week; 1=Used to drink, but have quit; 2=Drink 1–2 times a week; 3=Drink 3–4 times a week; 4=Drink every day | GRM | 0.57 (1.06) | 0.14 (0.58) | 0.35 (0.87) | <0.001 |
| 6 | What is your drinking habit? (If you do not currently drink, please answer based on your past drinking habits. One cup is equivalent to one bowl). | Ordinal | 4 | 0=Less than half a cup; 1=Half a cup to one cup; 2=Two to three cups; 3=Four or more cups | GRM | 0.77 (1.36) | 0.18 (0.71) | 0.47 (1.12) | <0.001 |
| 7 | How many years have you been drinking? (If you no longer drink, please answer based on your past experience.) | Ordinal | 5 | 0=Less than 1 year; 1=1-3 years; 2=3-5 years; 3=5-10 years; 4=More than 10 years | GRM | 1.10 (1.93) | 0.24 (0.94) | 0.66 (1.57) | <0.001 |
| 8 | Do you eat betel nut? | Ordinal | 5 | 0=Do not chew; 1=Used to chew, but have quit; 2=Chew 1-3 times a week; 3=Chew 4-5 times a week; 4=Chew 6 times a week or every day | GRM | 0.15 (0.54) | 0.01 (0.08) | 0.07 (0.39) | <0.001 |
| 9 | How many years have you been chewing betel nut? (If you have quit, please answer based on your past experience). | Ordinal | 6 | 0=Less than 1 year; 1=1 to less than 3 years; 2=3 to less than 5 years; 3=5 to less than 10 years; 4=10 to less than 15 years; 5=15 years or more | GRM | 0.10 (0.59) | 0.01 (0.06) | 0.04 (0.41) | <0.001 |
| 10 | On average, how many times do you chew betel nuts per day? (If you have quit, please answer based on your past experience). | Ordinal | 6 | 1=Less than 5; 2=6-10; 3=11-15; 4=16-20; 5=More than 21 | GRM | 0.07 (0.46) | 0.01 (0.49) | 0.37 (0.32) | <0.001 |
| 11 | For those who used to chew betel nut but have now quit, how long have you been abstaining from chewing betel nut? | Ordinal | 6 | 1=Less than 1 year; 2=1-3 years; 3=3-5 years; 4=5-10 years; 5=More than 10 years | GRM | 0.10 (0.59) | 0.01 (0.06) | 0.04 (0.41) | <0.001 |

Dichotomous items were modeled using the two-parameter logistic (2PL) model, whereas ordinal items were analyzed using the graded response model (GRM). For domains comprising both dichotomous and ordinal items, each item was modeled according to its response format within the same underlying latent construct.
We analyzed the data using the mean and standard deviation (SD) of all samples, including follow-up physical and clinical measurements across the entire cohort period. *p*-values were calculated using the Pearson chi-square test to assess differences between categorical variables.

**Supplementary Table 3.** Percentage distribution of missing data for all items selected for the Healthy Lifestyle and Behavioral Scale in the study cohort, based on 832,862 visits across the entire cohort.

| No. | Variable description | Structural missingness  n (%) | Intermittent missingness  n (%) |
| --- | --- | --- | --- |
| Sleep disturbance | | | |
| 1 | How many hours of sleep do you get every day? | 355,873 (42.7) | 58,673 (7.0) |
| 2 | How has the quality of your sleep been in the past month? | 164,200 (19.7) | 28,262 (3.4) |
| 3 | Have you had difficulty falling asleep in the past month? | 519,103 (62.3) | 189,792 (22.8) |
| 4 | Have you taken sleeping pills or sedatives to help you fall asleep in the past month? | 521,752 (62.6) | 190,268 (22.9) |
| 5 | During the past month, could you fall asleep but were easily awakened? | 515,536 (61.9) | 189,488 (22.8) |
| 6 | In the past month, have you had frequent dreams during sleep? | 517,984 (62.2) | 189,684 (22.8) |
| 7 | In the past month, would you say you often experienced deep sleep? | 513,480 (61.6) | 189,088 (22.7) |
| 8 | In the past month, did you often feel tired upon waking? | 514,564 (61.8) | 188,866 (22.7) |
| Physical inactivity | | | |
| 1 | How would you describe the intensity of your daily work activities? | 31,346 (3.76) | 11,730 (1.41) |
| 2 | What type of exercise do you do most often | 381,714 (45.83) | 166,666 (20.01) |
| 3 | What is your usual scheduled weekly time for exercise? | 349,101 (41.92) | 57,042 (6.85) |
| 4 | How many times have you exercised regularly in the past two weeks? | 397,251 (47.70) | 168,875 (20.28) |
| 5 | How much time do you usually spend exercising per session? | 343,652 (41.26) | 163,192 (19.59) |
| 6 | Are there any reasons why you cannot exercise? (e.g., health issues) | 487,403 (58.52) | 182,303 (21.89) |
| Suboptimal nutrition | | | |
| 1 | Do you eat at regular times and in regular amounts? | 95,822 (11.51) | 18,248 (2.19) |
| 2 | At what time do you usually have your final meal each day? | 556,749 (66.85) | 194,499 (23.35) |
| 3 | Do you usually take any nutritional supplements? | 25,553 (3.07) | 64,873 (7.79) |
| 4 | Are you a full-time vegetarian? | 13,896 (1.67) | 6,441 (0.77) |
| 5 | How frequently do you consume fried or pan-fried foods? (1 serving is about half a bowl). | 95,607 (11.48) | 19,533 (2.35) |
| 6 | How frequently do you consume fried, deep-fried, deep-fried, grilled, or smoked foods? | 556,765 (66.85) | 194,500 (23.35) |
| 7 | How much non-dark green vegetables do you usually eat (e.g., cabbage, bok choy, cucumber, radish, etc.) | 93,234 (11.19) | 17,436 (2.09) |
| 8 | How many servings of vegetables do you usually consume in a day? | 556,781 (66.85) | 194,500 (23.35) |
| 9 | How many servings of vegetables do you consume that are stir-fried with oil or prepared with salad dressing? | 374,626 (44.98) | 165,364 (19.85) |
| 10 | How many servings of fruit do you usually consume? (1 serving is about half medium of fruits such as apple, grapefruit, guava, etc.; 1 fruit such as orange, tangerine, kiwi, etc.; half a pound of fruits such as papaya, watermelon, etc.; 5 fruits such as lychees, etc.; 12 fruits such as grapes, longans, etc.) | 93,242 (11.20) | 17,510 (2.10) |
| 11 | Do you usually consume at least 2 servings of fruit per day? (1 serving is about 1 first size fruits such as apple, grapefruit, guava, orange, or tangerine, etc.; 1 palm-size of fruits such as papaya, watermelon, etc.; 1 handful of fruits such as lychees, grapes, etc.) | 556,791 (66.85) | 194,502 (23.35) |
| 12 | How many servings of rice or noodles do you usually consume? (1 serving is about 1 bowl of rice; 2 bowls of noodles or rice vermicelli; 4 thin slices of toast; one steamed bun; 2 pieces of pan-fried cakes/fried dough sticks) | 93,331 (11.21) | 17,530 (2.10) |
| 13 | What portion of your daily rice or noodle intake consists of who grains? (1 serving is about 4 slices of whole-wheat bread; 1 bowl of brown or multi-grain rice; 8 tablespoons of oatmeal). | 93,699 (11.25) | 17,836 (2.14) |
| 14 | How frequently do you choose germ rice, brown rice, whole-grain rice, or whole-wheat pasta? | 556,794 (66.85) | 194,504 (23.35) |
| 15 | What portion of your rice or noodle intake is fried or cooked with oil? (1 serving is about 1 bowl of fried rice, fried noodles, or fried rice noodles; or 2 pieces of pan-fried cakes or fried dough sticks). | 93,963 (11.28) | 17,843 (2.14) |
| 16 | How many servings of baked goods (such as sweet bread) do you usually eat? (1 serving is about 1 red bean bread, 1 crisp bread, 1 cake, or 15 sweet cookies). | 95,332 (11.45) | 19,204 (2.31) |
| 17 | How many servings of pickles, processed foods (such as ham, sausage, canned food, etc.), or salted fish do you usually eat? (1 serving is about one tablespoon of pickles, half a sausage, 2 slices of ham, or 1 tablespoon of canned meat). | 95,759 (11.50) | 19,537 (2.35) |
| 18 | How often do you eat instant noodles? (1 serving is about 1 bowl or 1 pack of instant noodles). | 95,609 (11.48) | 19,406 (2.33) |
| 19 | Do you usually dip your food in soy sauce or other salty condiments? (1 serving is about 2 teaspoons of soy sauce, ketchup, chili sauce, or black vinegar, or 1 teaspoon of salt and pepper). | 95,740 (11.50) | 19,409 (2.33) |
| 20 | How often do you dip your food in sauces or chili sauce? | 556,787 (66.85) | 194,504 (23.35) |
| 21 | When cooking, how often do you add extra soy sauce or other seasonings to enhance the flavor of food? | 556,803 (66.85) | 194,507 (23.35) |
| 22 | How often do you eat foods with jam or honey? (1 serving is about to about 2 teaspoons of jam or honey). | 350,552 (42.09) | 57,197 (6.87) |
| 23 | How often do you add extra sugar to your diet? (1 serving is about 2 teaspoons of white sugar, honey, jam, or fructose, or 3 sugar cubes). | 374,591 (44.98) | 165,357 (19.85) |
| 24 | How often do you eat sweet foods? | 556,808 (66.85) | 194,514 (23.35) |
| 25 | How often do you drink coffee, cocoa, tea, juice, or other beverages with added sugar (such as soda or sweetened mung bean soup)? (1 cup = 240 ml). | 95,496 (11.47) | 19,277 (2.31) |
| 26 | How often do you drink fresh fruit juice? | 556,798 (66.85) | 194,511 (23.35) |
| 27 | How much milk do you usually drink? (1 cup = 240 ml of fresh milk, 240 ml of yogurt, or 4 level tablespoons of milk powder). | 95,207 (11.43) | 18,143 (2.18) |
| 28 | How much dairy products do you usually eat? (1 serving is about 1 small cup of yogurt or 1 slice of or cheese). | 96,155 (11.55) | 18,711 (2.25) |
| 29 | How much beans and soy products do you usually eat? (1 serving is about half a box of tofu, 1 cup = 240 ml of soy milk, 2 blocks of five-spice dried tofu, or half a palm-sized piece of tofu) | 93,051 (11.17) | 17,331 (2.08) |
| 30 | How much seafood do you usually eat? (1 serving is about 1 medium-sized saury, 4 slices of sashimi, 4 tablespoons of cooked fish, 4 grass shrimp, or 16 oysters). | 103,012 (12.37) | 20,360 (2.44) |
| 31 | How many eggs do you usually eat? (1 serving is about 1 chicken or duck egg, or 5 pigeon eggs) | 96,029 (11.53) | 18,270 (2.19) |
| 32 | How much meat do you usually eat (such as pork, chicken, duck, lamb, or beef)? (1 plate is about 1 slice of pork steak [palm-sized, 1 cm thick], 1 pork shank leg, 1 hamburger patty, or 4 tablespoons of other lean meat). | 103,058 (12.37) | 20,423 (2.45) |
| 33 | How much offal do you usually eat (such as liver, kidney, heart, intestines, etc.)? (1 serving is about half a bowl of pork or chicken liver, 8 chicken hearts, or 4 tablespoons of cooked pork intestines). | 103,331 (12.41) | 20,482 (2.46) |
| Substance use | | | |
| 1 | Do you smoke? | 583,906 (70.11) | 126,281 (15.16) |
| 2 | How many years have you been smoking? | 535,083 (64.25) | 110,629 (13.28) |
| 3 | How many cigarettes do you usually smoke per day? (If you have quit, please answer based on your past smoking habits). | 594,752 (71.41) | 143,137 (17.19) |
| 4 | On average, how many cigarettes do you smoke per day? (If you have quit, please answer based on your past experience). | 584,277 (70.1) | 126,779 (15.2) |
| 5 | Do you drink alcohol? | 109,917 (13.20) | 23,430 (2.81) |
| 6 | What is your drinking habit? (If you do not currently drink, please answer based on your past drinking habits. One cup is equivalent to one bowl). | 100,457 (12.06) | 22,338 (2.68) |
| 7 | How many years have you been drinking? (If you no longer drink, please answer based on your past experience.) | 104,318 (12.53) | 23,923 (2.87) |
| 8 | Do you eat betel nut? | 39,289 (4.72) | 18,662 (2.24) |
| 9 | How many years have you been chewing betel nut? (If you have quit, please answer based on your past experience). | 55,925 (6.71) | 27,477 (3.30) |
| 10 | On average, how many times do you chew betel nuts per day? (If you have quit, please answer based on your past experience). | 54,299 (6.52) | 27,316 (3.28) |
| 11 | For those who used to chew betel nut but have now quit, how long have you been abstaining from chewing betel nut? | 55,925 (6.71) | 27,477 (3.30) |

**Supplementary Table 4.** Item loadings and discrimination parameters of the healthy lifestyle and behavior scale

| No. | Variable description | EFA factor loading | IRT discrimination | CFA standardized loading |  |
| --- | --- | --- | --- | --- | --- |
| Sleep disturbance | | | | | |
| 1 | Have you had difficulty falling asleep in the past month? | 0.56 | 0.52 | 0.50 |  |
| 2 | Have you taken sleeping pills or sedatives to help you fall asleep in the past month? | 0.54 | 0.54 | 0.51 |  |
| 3 | During the past month, could you fall asleep but were easily awakened? | 0.70 | 0.62 | 0.55 |  |
| 4 | In the past month, have you had frequent dreams during sleep? | 0.40 | 0.40 | 0.41 |  |
| 5 | In the past month, would you say you often experienced deep sleep? | 0.85 | 0.80 | 0.83 |  |
| Physical inactivity | | | | | |
| 1 | What type of exercise do you do most often? | 0.41 | 0.42 | 0.40 |  |
| 2 | How many times have you exercised regularly in the past two weeks? | 0.40 | 0.40 | 0.40 |  |
| 3 | How much time do you usually spend exercising per session? | 0.89 | 0.84 | 0.88 |  |
| Suboptimal nutrition | | | | | |
| 1 | How frequently do you consume fried or pan-fried foods? (1 serving is about half a bowl). | 0.68 | 0.72 | 0.70 |  |
| 2 | What portion of your rice or noodle intake is fried or cooked with oil? (1 serving is about 1 bowl of fried rice, fried noodles, or fried rice noodles; or 2 pieces of pan-fried cakes or fried dough sticks). | 0.51 | 0.51 | 0.51 |  |
| 3 | How many servings of pickles, processed foods (such as ham, sausage, canned food, etc.), or salted fish do you usually eat? (1 serving is about one tablespoon of pickles, half a sausage, 2 slices of ham, or 1 tablespoon of canned meat). | 0.68 | 0.69 | 0.68 |  |
| 4 | How often do you eat instant noodles? (1 serving is about 1 bowl or 1 pack of instant noodles). | 0.49 | 0.51 | 0.48 |  |
| 5 | Do you usually dip your food in soy sauce or other salty condiments? (1 serving is about 2 teaspoons of soy sauce, ketchup, chili sauce, or black vinegar, or 1 teaspoon of salt and pepper). | 0.60 | 0.60 | 0.59 |  |
| 6 | How often do you add extra sugar to your diet? (1 serving is about 2 teaspoons of white sugar, honey, jam, or fructose, or 3 sugar cubes). | 0.51 | 0.52 | 0.52 |  |
| 7 | How often do you drink coffee, cocoa, tea, juice, or other beverages with added sugar (such as soda or sweetened mung bean soup)? (1 cup = 240 ml). | 0.51 | 0.49 | 0.51 |  |
| 8 | How many eggs do you usually eat? (1 serving is about 1 chicken or duck egg, or 5 pigeon eggs) | 0.45 | 0.45 | 0.46 |  |
| 9 | How much meat do you usually eat (such as pork, chicken, duck, lamb, or beef)? (1 plate is about 1 slice of pork steak [palm-sized, 1 cm thick], 1 pork shank leg, 1 hamburger patty, or 4 tablespoons of other lean meat). | 0.48 | 0.52 | 0.53 |  |
| 10 | How much offal do you usually eat (such as liver, kidney, heart, intestines, etc.)? (1 serving is about half a bowl of pork or chicken liver, 8 chicken hearts, or 4 tablespoons of cooked pork intestines). | 0.55 | 0.57 | 0.56 |  |
| Substance use | | | | | |
| 1 | Do you smoke? | 0.98 | 0.77 | 0.98 |  |
| 2 | How many years have you been smoking? | 0.98 | 0.70 | 0.98 |  |
| 3 | How many cigarettes do you usually smoke per day? (If you have quit, please answer based on your past smoking habits). | 0.96 | 0.76 | 0.98 |  |
| 4 | On average, how many cigarettes do you smoke per day? (If you have quit, please answer based on your past experience). | 0.97 | 0.80 | 0.98 |  |
| 5 | Do you drink alcohol? | 0.55 | 0.97 | 0.94 |  |
| 6 | What is your drinking habit? (If you do not currently drink, please answer based on your past drinking habits. One cup is equivalent to one bowl). | 0.57 | 0.98 | 0.96 |  |
| 7 | How many years have you been drinking? (If you no longer drink, please answer based on your past experience.) | 0.59 | 0.98 | 0.98 |  |
| 8 | Do you eat betel nut? | 0.66 | 0.70 | 0.71 |  |

EFA, Exploratory Factor Analysis; IRT, Item Response Theory; CFA, Confirmatory Factor Analysis.

**Supplementary Table 5.** Confirmatory factor analysis fit indices for healthy lifestyle and behavioral components

| Components | CFI | TLI | RMSEA | SRMR |
| --- | --- | --- | --- | --- |
| Sleep disturbance | 0.975 | 0.950 | 0.055 | 0.066 |
| Physical inactivity | 0.980 | 0.970 | 0.055 | 0.077 |
| Suboptimal nutrition | 0.946 | 0.930 | 0.072 | 0.074 |
| Substance use | 0.966 | 0.950 | 0.064 | 0.057 |

CFI, Comparative Fit Index (acceptable fit >0.90, good fit >0.95); TLI, Tucker-Lewis Index (acceptable fit >0.90, good fit >0.95); RMSEA, Root Mean Square Error of Approximation (close fit <0.05, reasonable fit 0.05–0.08); SRMR, Standardized Root Mean Square Residual (acceptable fit <0.05).

**Supplementary Table 6.** Selected updated questionnaire items for sensitivity analysis

| No. | Variable description | Coding | Date modified |
| --- | --- | --- | --- |
| Sleep disturbance | | | |
| 1 | Have you had difficulty falling asleep in the past month? | 0=No; 1=Yes | 2014 - 2023 |
| 2 | Have you taken sleeping pills or sedatives to help you fall asleep in the past month? | 0=No; 1=Yes | 2014 - 2023 |
| 3 | During the past month, could you fall asleep but were easily awakened? | 0=No; 1=Yes | 2014 - 2023 |
| 4 | In the past month, have you had frequent dreams during sleep? | 0=No; 1=Yes | 2014 - 2023 |
| 5 | In the past month, would you say you often experienced deep sleep? | 0=No; 1=Yes | 2014 - 2023 |
| Physical inactivity | | | |
| 1 | Do you often perform anaerobic exercises, such as moderate-weight training, strength training, or push-ups? | 0=No; 1=Yes | 2017 - 2023 |
| 2 | Do you often do stretching exercises, such as light yoga, Tai Chi, or general stretching? | 0=No; 1=Yes | 2017 - 2023 |
| 3 | Do you often do mild aerobic exercises, such as walking or cycling? | 0=No; 1=Yes | 2017 - 2023 |
| 4 | Do you often engage in moderate aerobic exercise, such as brisk walking or mountain climbing? | 0=No; 1=Yes | 2017 - 2023 |
| 5 | Do you often engage in heavy aerobic exercise, such as swimming or running? | 0=No; 1=Yes | 2017 - 2023 |
| 6 | Do you often engage in intense aerobic exercise, such as skipping or rowing? | 0=No; 1=Yes | 2017 - 2023 |
| 7 | How many times have you exercised regularly in the past two weeks? | 0=Every day; 1=4–6 times per week; 2=2–3 times per week; 3=Once per week; 4= No exercise or less than once per week | 2017 - 2023 |
| Suboptimal nutrition | | | |
| 1 | How frequently do you consume fried or pan-fried foods? (1 serving is about half a bowl). | 0=None or less than once per week; 1=2–3 times per week; 2=4–5 times per week; 3=6 times per week or more / daily | 2017 - 2023 |
| 2 | How frequently do you choose germ rice, brown rice, whole-grain rice, or whole-wheat pasta? | 0=Never; 1=Occasionally; 2=Often; 3=Always | 2017 - 2023 |
| 3 | How many servings of pickles, processed foods (such as ham, sausage, canned food, etc.), or salted fish do you usually eat? (1 serving is about one tablespoon of pickles, half a sausage, 2 slices of ham, or 1 tablespoon of canned meat). | 0=Never; 1=Occasionally; 2=Often; 3=Always | 2017 - 2023 |
| 4 | Do you usually dip your food in soy sauce or other salty condiments? (1 serving is about 2 teaspoons of soy sauce, ketchup, chili sauce, or black vinegar, or 1 teaspoon of salt and pepper). | 0=Never; 1=Occasionally; 2=Often; 3=Always | 2017 - 2023 |
| 5 | How often do you add extra sugar to your diet? (1 serving is about 2 teaspoons of white sugar, honey, jam, or fructose, or 3 sugar cubes). | 0=None or less than once per week; 1=2–3 times per week; 2= 4–5 times per week; 3= 6 times per week or more / daily | 2017 - 2023 |
| 6 | How often do you drink fresh fruit juice? | 0=Never; 1=Occasionally; 2=Often; 3=Always | 2017 - 2023 |
| 7 | How much dairy products do you usually eat? (1 serving is about 1 small cup of yogurt or 1 slice of or cheese). | 0=No or less than 1 serving per week; 1=1-3 servings per week; 2=4-6 servings per week; 3=1 serving per day; 4=2 or more servings per day | 2017 - 2023 |
| Substance use | | | |
| 1 | Do you smoke? | 0=Never smoked; 1=Do not smoke but are frequently exposed to secondhand smoke; 2=Former smoker (used to smoke but quit); 3=Occasional smoker; 4=Daily smoker | 2003 - 2023 |
| 2 | How many years have you been smoking? | 0=Less than 1 year; 1=1–3 years; 2=3–10 years; 3=10–20 years; 4=More than 20 years | 2009 - 2023 |
| 3 | How many cigarettes do you usually smoke per day? (If you have quit, please answer based on your past smoking habits). | 0=Less than half a pack per day; 1=Half a pack to one pack per day; 2=More than one pack per day | 2003 - 2023 |
| 4 | On average, how many cigarettes do you smoke per day? (If you have quit, please answer based on your past experience). | 0= Do not smoke; 1=Less than 5; 2=6-10 per day (about half a pack); 3= 11-20 per day (about one pack); 4=More than 20 per day (more than one pack) | 2003 - 2023 |
| 5 | Do you drink alcohol? | 0=No or less than one drink; 1=Former drinker (quit drinking); 2=1 to 2 drinks per week; 3=3 to 4 drinks per week; 4=5 to 6 drinks per week; 5=Daily drinking |  |
| 6 | What is your drinking habit? (If you do not currently drink, please answer based on your past drinking habits. One cup is equivalent to one bowl). | 0=Less than half a cup; 1=Half a cup to one cup; 2=Two to three cups; 3=Four or more cups | 2000 - 2023 |
| 7 | How many years have you been drinking? (If you no longer drink, please answer based on your past experience.) | 0=Less than 1 year; 1=1 to less than 3 years; 2=3 to less than 5 years; 3=5 to less than 10 years; 4=10 to less than 15 years; 5=15 years or more | 2000 - 2023 |
| 8 | Do you eat betel nut? | 0=Do not chew; 1=Used to chew, but have quit; 2=Chew 1-3 times a week; 3=Chew 4-5 times a week; 4=Chew 6 times a week or every day | 2000 - 2023 |

The modified questionnaire items included in this table were the final items selected for the sensitivity analysis, as they demonstrated higher variance explained by each component: sleep disturbance increased from 40.0% to 50.0%, physical inactivity from 25.8% to 50.2%, suboptimal nutrition from 20.2% to 53.7%, and substance use from 23.0% to 66.3%.

**Supplementary Table 7.** Sensitivity analysis of the adjusted association between HLBS and the risk of MetS development using updated questionnaire items (n= 21,240 participants)

| Variables | RR | 95% CI | | p-value |
| --- | --- | --- | --- | --- |
|  |  | Lower | Upper |  |
| *HLBS^* |  |  |  |  |
| High | 1 |  |  |  |
| Moderate | 1.164 | 1.096 | 1.237 | <0.001 |
| Low | 1.581 | 1.249 | 1.637 | <0.001 |
| *HLBS components* |  |  |  |  |
| Sleep disturbance | 1.150 | 1.122 | 1.168 | <0.001 |
| Physical inactivity | 1.014 | 1.003 | 1.055 | <0.001 |
| Suboptimal nutrition | 1.025 | 1.021 | 1.028 | <0.001 |
| Substance used | 1.050 | 1.042 | 1.058 | <0.001 |

^p-values trend < 0.005.
HLBS, health lifestyle and behavioral scale; MetS, metabolic syndrome; RR, relative risk; CI, confidence interval.
For the HLBS analysis, we adjusted for age, sex, education, household annual income, and calendar year. For each HLBS component, we additionally adjusted for sleep quality, physical activity, nutritional intake, and substance use, excluding the component that was being analyzed in the model.
We conducted a sensitivity analysis using the modified questionnaire items, restricting the analysis to the period when most items had already been updated (2017 to 2023), to ensure greater consistency and comparability of measurements across participants and to minimize variability in item distributions across survey years.

*Appendix a.* We collected items corresponding to the healthy lifestyle and behavior components from the MJ Health database. Items related to sleep disturbance were derived from the 21-item Sleep Quality Questionnaire. Physical inactivity was assessed using the 47-item Exercise Habits Questionnaire. Suboptimal nutrition was measured using the 123-item Eating Habits Questionnaire, which comprised 43 general eating habit items, 19 fat intake items, 26 fruit, vegetable, fiber, and antioxidant items, 9 salt intake items, 3 water intake items, 13 refined sugar and dairy intake items, and 10 bean, fish, meat, and egg items. Substance use was measured using the 38-item Lifestyle Habits Questionnaire, which included 12 smoking habit items, 14 drinking habit items, 6 betel nut chewing habit items, and 6 oral hygiene habit items.

*Appendix b**.* Certain questionnaire item responses corresponding to specific HLBS pillars were modified over time to improve measurement precision. Sleep duration was initially assessed using a single item, “How much sleep do you get every day?”, administered from 2000 to 2008, with four response categories: (1) 0 to 4 hours (revised in 2007 to “less than 4 hours”), (2) 4 to 6 hours, (3) 6 to 8 hours, and (4) more than 8 hours. Beginning in 2009, this item was revised to “How long do you sleep on average during each sleep period?”, with expanded response options: (1) less than 4 hours, (2) 4 to 6 hours, (3) 6 to 7 hours, (4) 7 to 8 hours, (5) 8 to 9 hours, and (6) more than 9 hours, allowing for a more granular assessment of sleep duration. Similarly, the sleep quality item “How was your sleep?” was later refined to “How was your sleep in the past month?” to introduce a defined recall period.

For items related to physical activity, the questionnaire initially included a single item used from 2009 to 2016: “What kind of exercise do you do most often?”, with response categories defined as (0) vigorous exercise (e.g., gym lifting, running, skipping), (1) heavy exercise (e.g., jogging, climbing), (2) moderate exercise (e.g., playing sports, swimming), (3) light exercise (e.g., brisk walking, cycling), and (4) no exercise. In later survey waves, starting in 2017, this item was modified into multiple specific questions assessing different types of physical activity, including anaerobic exercise, stretching exercises (e.g., yoga, Tai Chi), mild aerobic activity (e.g., walking, cycling), moderate aerobic activity (e.g., brisk walking, mountain climbing), heavy aerobic activity (e.g., swimming, running), and intense aerobic activity (e.g., skipping, rowing), each with binary (Yes/No) response options.

For nutritional status, most items were refined beginning in 2017 to improve measurement detail. For example, the item “Do you eat at regular times and in regular amounts?” (Yes/No) was replaced with “At what time do you usually have your last meal of the day?”, with response options: (1) before 5:00 PM, (2) 5:00 PM to 6:00 PM, (3) 6:00 PM to 7:00 PM, (4) 7:00 PM to 8:00 PM, (5) 8:00 PM to 9:00 PM, and (6) after 9:00 PM. Similarly, the item “How frequently do you consume fried or pan-fried foods? (one serving = half a bowl)” with responses ranging from 0 = no or less than 1 serving per week to 4 = 2 or more servings per day, was expanded to “How frequently do you consume fried, deep-fried, grilled, or smoked foods?”, with revised response options: 0 = no or less than 1 serving per week, 1 = 2 to 3 servings per week, 2 = 4 to 5 servings per week, and 3 = 6 servings per week or every day.

For substance use, smoking status was initially assessed using the item “Do you smoke?” with response options: (1) rarely smoke, (2) used to smoke but have quit, (3) occasionally smoke, (4) smoke frequently, and (5) smoke every day. After 2002, the response options were expanded and refined to improve clarity and structure, including (1) no smoking, (2) do not smoke but often inhale secondhand smoke, (3) used to smoke but have quit, (4) smoke 1 to 3 times per week, (5) smoke 4 to 5 times per week, and (6) smoke every day. Similarly, one item related to drinking status was modified in 2016. The original question, “Do you drink alcohol?”, included response options: (1) rarely drink, (2) used to drink but have quit, (3) occasionally drink, (4) drink frequently, and (5) drink every day. This was later refined to improve clarity and response flow, with revised options: (1) do not drink or drink very little, (2) used to drink but have quit, (3) drink 1 to 2 times per week, (4) drink 3 to 4 times per week, and (5) drink every day.

To ensure comparability across questionnaire waves, these items were harmonized by mapping the revised activity-specific responses to the original categorical framework, aligning conceptually equivalent activity levels and constructing unified categories for analysis across all samples.

*Appendix c.* The selected items were evaluated for content validity to confirm that they appropriately reflected the intended constructs. Each item’s relevance to the overarching construct (pillar) was assessed using evidence from prior studies. Items deemed irrelevant to the defined pillars were subsequently removed. Examples include “Have you felt down and unmotivated in the past two weeks?” for the sleep quality dimension, and “Does your mouth bleed when you brush your teeth?” or “Do your teeth feel a little loose?” for the substance use dimension. Items that were too narrow or redundant were also removed to avoid overlap, such as detailed questions like “Which particular meal or snack do you usually eat for breakfast, lunch, or dinner?”, repeated questions such as “How much fried or pan-fried food should be eaten?” in the nutrition dimension, and “What kind of exercise do you most often do?” in the physical inactivity dimension. Items that were too broad or vague were excluded as well, such as “How much energy did you have in the past four weeks?” in the sleep quality dimension.

*Appendix d*. To support the missing data framework, we first identified the baseline item instruments, which included sleep disturbance (21-item scale), physical inactivity (47-item scale), suboptimal nutrition (123-item scale), and substance use (38-item scale). Full information on the list of available items is provided in the MJ Health Questionnaire Database (<https://www.mjhrf.org/index.php?action=database&id=4>). Auxiliary variables were then derived from baseline lifestyle instruments aligned with the lifestyle medicine pillars proposed by Frates et al. (2021) [1]. From these instruments, items conceptually aligned with the study framework were identified and retained, resulting in 8 items for sleep disturbance, 6 items for physical inactivity, 33 items for suboptimal nutrition, and 11 items for substance use. These selected items are detailed in Supplementary Table 3. Using these variables, we first conducted. Using the “psych” package in R version 4.5.1, we first conducted the exploratory factor analysis (EFA), excluding participants with excessive missing responses, as standard EFA cannot accommodate incomplete data [2]. This ensured that factor estimation relied on participants with sufficient responses. We then performed the confirmatory factor analysis (CFA) using the “lavaan” package in R, with models estimated through full information maximum likelihood (FIML). FIML accounts for intermittent missingness under the missing-at-random assumption by utilizing all available responses from each participant, allowing partial data to contribute to parameter estimation [3, 4]. Although some missingness in our cohort is structural, arising from changes in questionnaire versions over time, it is largely predictable from observed study design variables (e.g., visit date, questionnaire version). By incorporating these variables into the modeling framework, FIML provides consistent and asymptotically unbiased estimates despite structural missingness.

*Appendix e.* Before performing the analysis, we reverse-coded five physical activity items to ensure their alignment with the other healthy lifestyle and behavior components. Performing the exploratory factor analysis (EFA), factor solutions were extracted using maximum likelihood estimation with oblimin rotation. Factor loadings also allowed calculation of the proportion of variance explained by each factor, reflecting each factor’s contribution to the overall construct [2]. The selection of the best-fit items was guided by an EFA, in which items with factor loadings below 0.30 were considered for removal, while all retained items loaded above 0.40, indicating strong representation of the latent constructs [2]. For the confirmatory factor analysis (CFA), model fit was evaluated using standard indices, including a comparative fit index (CFI) > 0.90, Tucker–Lewis index (TLI) > 0.90, root mean square error of approximation (RMSEA) < 0.05, and standardized root mean square residual (SRMR) < 0.08 [3, 4].

**References**

1.	Frates B, Bonnet J, Joseph R, et al. (2018) Lifestyle medicine handbook: An introduction to the power of healthy habits. Healthy learning. 10: 1606794132

2.	Tavakol M and Wetzel A Factor analysis: A means for theory and instrument development in support of construct validity. Int J Med Educ.(2020); 11: 245-247

3.	Goretzko D, Siemund K, and Sterner P Evaluating model fit of measurement models in confirmatory factor analysis. Educ Psychol Meas.(2024); 84(1): 123-144

4.	Brown TA Confirmatory factor analysis for applied research, 2nd ed. in Confirmatory factor analysis for applied research, 2nd ed. New York, NY, US: The Guilford Press.
